# Metacognitive therapy home-based self-help for anxiety and depression in cardiovascular disease patients in the UK: A single-blind randomised controlled trial

**DOI:** 10.1371/journal.pmed.1004161

**Published:** 2023-01-31

**Authors:** Adrian Wells, David Reeves, Calvin Heal, Peter Fisher, Patrick Doherty, Linda Davies, Anthony Heagerty, Lora Capobianco

**Affiliations:** 1 Faculty of Biology, Medicine and Health, School of Psychological Sciences, The University of Manchester, Manchester, United Kingdom; 2 Research and Innovation, Greater Manchester Mental Health NHS Foundation Trust, Manchester, United Kingdom; 3 NIHR School for Primary Care Research, Manchester Academic Health Science Centre, The University of Manchester, Manchester, United Kingdom; 4 Institute of Psychology, Health and Society, University of Liverpool, Liverpool, United Kingdom; 5 Liverpool Clinical Health, The Royal Liverpool and Broadgreen University Hospital NHS Trust, Liverpool, United Kingdom; 6 Department of Health Sciences, University of York, York, United Kingdom; 7 Centre for Health Economics, Division of Population Health, Health Services Research and Primary Care, Faculty of Biology Medicine and Health, School of Health Sciences, The University of Manchester, Manchester, United Kingdom; 8 Core Technology Facility, The University of Manchester School of Medical Sciences, Manchester, United Kingdom; 9 Manchester University NHS Foundation Trust, Manchester Royal Infirmary, Manchester, United Kingdom; Stellenbosch University, SOUTH AFRICA

## Abstract

**Background:**

Anxiety and depression in cardiac rehabilitation (CR) are associated with greater morbidity, mortality, and increased healthcare costs. Current psychological interventions within CR have small effects based on low-quality studies of clinic-based interventions with limited access to home-based psychological support. We tested the effectiveness of adding self-help metacognitive therapy (Home-MCT) to CR in reducing anxiety and depression in a randomised controlled trial (RCT).

**Methods and findings:**

We ran a single-blind, multi-centre, two-arm RCT. A total of 240 CR patients were recruited from 5 NHS-Trusts across North West England between April 20, 2017 and April 6, 2020. Patients were randomly allocated to Home-MCT+CR (*n* = 118, 49.2%) or usual CR alone (*n* = 122, 50.8%). Randomisation was 1:1 via randomised blocks within hospital site, balancing arms on sex and baseline Hospital Anxiety and Depression Scale (HADS) scores. The primary outcome was the HADS total score at posttreatment (4-month follow-up). Follow-up data collection occurred between August 7, 2017 and July 20, 2020. Analysis was by intention to treat. The 4-month outcome favoured the MCT intervention group demonstrating significantly lower end of treatment scores (HADS total: adjusted mean difference = −2.64 [−4.49 to −0.78], *p* = 0.005, standardised mean difference (SMD) = 0.38). Sensitivity analysis using multiple imputation (MI) of missing values supported these findings. Most secondary outcomes also favoured Home-MCT+CR, especially in reduction of post-traumatic stress symptoms (SMD = 0.51). There were 23 participants (19%) lost to follow-up in Home-MCT+CR and 4 participants (3%) lost to follow-up in CR alone. No serious adverse events were reported. The main limitation is the absence of longer term (e.g., 12-month) follow-up data.

**Conclusion:**

Self-help home-based MCT was effective in reducing total anxiety/depression in patients undergoing CR. Improvement occurred across most psychological measures. Home-MCT was a promising addition to cardiac rehabilitation and may offer improved access to effective psychological treatment in cardiovascular disease (CVD) patients.

**Trial registration:**

NCT03999359.

## Abstract 2

In a randomized controlled trial, Adrian Wells and colleagues test the effectiveness of adding self-help metacognitive therapy to cardiac rehabilitation in reducing anxiety and depression.

## Author summary

**Why was this study done?:**

- One-third of cardiovascular disease (CVD) patients suffer anxiety and depression symptoms, but psychological support in cardiac rehabilitation (CR) is limited and produces small effects.
- Treating anxiety and depression is important because they are associated with worse health outcomes and impair quality of life.
- Previously, a recent treatment called metacognitive therapy (MCT) significantly improved anxiety and depression in patients undergoing CR when delivered by cardiac staff.
- Home-based self-help MCT was previously found to be feasible and acceptable to deliver alongside CR, hence a full-scale trial was required to test the effectiveness of this self-help intervention.

**What did the researchers do and find?:**

- We used a single-blind parallel randomised trial to test the effectiveness of self-help MCT.
- A total of 240 CR patients with elevated anxiety and/or depression received either usual CR or usual CR plus self-help MCT.
- The addition of self-help MCT significantly improved anxiety and depression (Hospital Anxiety and Depression Scale (HADS)) compared to CR alone (standardised mean difference (SMD) = 0.38). In CR alone, 36% of patients met criteria for minimal clinically important improvement, compared to 59% achieving this status under MCT self-help.
- MCT self-help plus CR was also found to improve post-traumatic stress symptoms to a greater extent than CR alone. An unexpected finding was that MCT self-help was also associated with a reduced rate in worsening of anxiety and depression during CR.

**What do these findings mean?:**

- Anxiety and depression in CVD can be effectively managed with self-help MCT.
- Self-help MCT has the potential in the future to improve health outcomes, reduce the risk of psychological deterioration in CVD, and improve access to evidence-based psychological care.
- The current findings do not address long-term outcomes, but they support a future policy of managing the psychological needs of patients undergoing CR with a menu of MCT options that includes home-based self-help.

## Introduction

Cardiovascular disease (CVD) is the leading cause of death globally [1]. However, survival rates are increasing with approximately 550 million people living with heart and circulatory disease [1]. To support recovery from cardiovascular events, cardiac rehabilitation (CR) is recommended [2,3]. CR is primarily delivered using a centre-based approach; however, home-based programmes are also offered to increase patient choice and engagement and are equally effective [4]. CR has been shown to be cost-effective, reduce hospital admissions and mortality, and increase physical activity and psychological well-being [5–7].

While CR improves anxiety and depression, the effects are small, limiting the benefits that CR alone might have on improving psychological health. This is important because anxiety and depression are common among heart disease patients [8], are associated with decreased attendance at CR, increased healthcare costs, further cardiac events, and increased mortality [5–7,9–10]. As such, it is important to effectively recognise and treat anxiety and depression in CR services to improve clinical outcomes, quality of life, and potentially reduce healthcare costs.

While CR services offer home and centre-based CR programmes that are exercise and lifestyle based, less emphasis has been placed on offering effective home-based/self-help psychological support for CR patients. When home-based psychological therapies are offered, cognitive behavioural therapy (CBT) techniques are used but the effectiveness is limited [11,12]. Matcham and colleagues’ [12] systematic review and meta-analysis of 25 studies evaluating self-help interventions for symptoms of depression, anxiety, and distress in patients with physical illnesses found only 4 focused on cardiac patients (*n* = 678) [12]. Interventions in these studies consisted of stress management and relaxation, while control groups included standard care which was comprised of educational sessions and counselling with a nurse. Results across the 4 studies are mixed and the sample characteristics vary from pre-operative patients [13], angina sufferers [14,15], and post-MI rehabilitation [16]. In most of the studies, anxiety and depression were secondary outcomes, patients with a history of psychiatric problems were excluded and baseline psychological distress levels were low. Such factors may contribute to the small or nonsignificant effects observed in anxiety or depression improvement. More recent randomised controlled trials (RCTs) have evaluated internet CBT (iCBT) for CVD patients with mixed results [17,18]. However, such studies involve therapist-led interventions that differ from self-help. In summary, the current evidence on self-help is limited, samples are heterogenous, psychological outcomes are usually secondary, and the effects on anxiety and depression are small and inconsistent. Furthermore, most treatments have used stress management methods (e.g., relaxation, challenging misconceptions), which may not fit particularly well with the needs of all CVD patients [19,20]. The status of the field indicates a need for adequately controlled and suitably powered trials of psychological treatments delivered as self-help and based on models that target causal mechanisms of anxiety and depression.

A candidate treatment approach is metacognitive therapy (MCT [21]), which is a theory-based, transdiagnostic psychological therapy that has been shown to be effective in treating anxiety and depression in mental health settings [22]. MCT may be particularly well suited to most CR patients because it helps patients to discover effective ways of regulating repetitive negative thinking such as worry and rumination, without the need to challenge varied individual concerns, which can be realistic. Furthermore, there is evidence that MCT can work in CVD patients. Wells and colleagues [23] conducted a large-scale randomised trial of therapist delivered MCT in a group-based format for CVD patients attending CR. When group-MCT was added to usual CR, it was found to be more effective than usual CR alone in reducing symptoms of anxiety and depression on Hospital Anxiety and Depression Scale (HADS) total at posttreatment (standardised mean difference (SMD) = 0.52) and at 12 months follow-up (SMD = 0.33). The results open-up the possibility that MCT delivered in other formats may also be effective, but this has yet to be evaluated. In particular, a self-help, home-based version of MCT could extend effective delivery and increase access to psychological support in CR, meeting a core objective of the British Association for Cardiovascular Prevention and Rehabilitation (BACPR), of providing greater patient choice [2].

The present study is to our knowledge, the first large-scale trial of a self-help, home-based MCT in patients with CVD. We evaluated if the addition of home-MCT to usual cardiac rehabilitation improved anxiety and depression. The trial tested the primary hypothesis that the addition of home-MCT to usual CR is more effective than CR alone in alleviating symptoms of anxiety and depression in patients with CVD between baseline and 4-month follow-up. We set a minimal inclusion criterion as presence of mild symptoms of anxiety, depression, or both, based on HADS scores, which could include individuals with current or previous psychological disorders.

## Methods

### Study design

A multicentre two-arm, single-blind randomised, controlled trial with 4-month follow-up comparing home-MCT plus usual CR (Home MCT+CR) with usual CR alone was conducted between April 2017 and March 2020. Patients were recruited between April 20, 2017 and April 6, 2020. Follow-up data collection occurred between August 7, 2017 and July 20, 2020. Patients were recruited from CR services at 5 National Health Service (NHS) hospital trusts (Aintree University Hospitals NHS Foundation Trust, Bolton NHS Foundation Trust, Manchester University NHS Foundation Trust, East Cheshire NHS Trust, and Pennine Acute Hospitals NHS Trust) across the North West of England. Ethical approval was obtained from the North West—Greater Manchester West Research Ethics Committee (REC Reference 16/NW/0786), along with site-specific approval. The trial is registered with clinicaltrials.gov, NCT No. NCT03129282 and No. NCT03999359. The study began as a feasibility and acceptability trial (NCT No. NCT03129282) that was conducted April 2017 to June 2020). The trial was then granted a variation to contract (VTC) by the funder in March 2019 to progress from a pilot feasibility study (*n* = 108) to a definitive RCT. The trial registration for the progression to a definitive trial can be found here: NCT No. NCT03999359. The definitive RCT was conducted between May 2019 and August 2020. The results of the feasibility study are reported elsewhere [24]. The VTC provided for further recruitment and collection of baseline and 4-month follow-up data, but not 12-month follow-up which was beyond the project resources. No changes were made to the trial procedures or measures following the feasibility study and the data was not unblinded. To assess if we were able to progress from feasibility to full-scale trial, analyses were conducted to assess the rates of missing data and variability in outcome measures, which indicated low rates of missing data and sufficient variability to the data to detect change. We employed the following strategy to extend data collection: (1) all analysis was conducted without breaking the blinding of the PI or trial statisticians; (2) no separate trial-arm analysis was undertaken; and (3) none of the 4- or 12-month outcome data was examined, except for 4-month completion rates. Consequently, no estimate of the effectiveness of Home-MCT was made from the data collected under the pilot since our aim was to extend data collection and use the pilot as an internal-pilot study. Therefore, with the approval of the funders and Trial Steering Committee, all data was pooled for the present RCT analysis. The study is presented in line with CONSORT guidelines [25]. A separate protocol was not published for the extension to a full-scale trial as the study followed the published protocol for the feasibility trial [26] to which no changes were made apart from extending recruitment.

### Participants

The trial included participants referred to CR services who met the Department of Health or British Association for Cardiac Prevention and Rehabilitation CR eligibility. As part of the CR program, all patients referred to CR are sent a National Audit of Cardiac Rehabilitation assessment pack [2], which includes a HADS questionnaire [27] to be returned to the CR team at CR assessment. Patients who had a score of 8 or greater on either the depression or anxiety subscale of the HADS [27] were aged 18 years or older and had a competent level of English language comprehension were eligible to take part. For more details on the inclusion/exclusion criteria, see the study protocol [26]. A score of 8 or greater is considered to be the cutoff for mild clinical symptoms and yields the optimal sensitivity and specificity for identifying clinical caseness [28]. Reasons for referral to CR by group are summarised in Table 1. Patients who scored 8 or above on the anxiety or depression subscale of the HADS were screened for eligibility by the CR staff. Eligible patients were provided with study information and contacted by a research assistant (RA) to obtain written or verbal consent and administer baseline questionnaires before starting CR.

**Table 1:** Baseline demographic and clinical characteristics.

	Home-MCT plus CR (*n* = 118)	CR alone (*n* = 122)
Age	60.0 (10.3)	61.2 (10.8)
Sex		
Male	74 (62.7%)	71 (58.2%)
Female	44 (37.3%)	51 (41.8%)
Hospital site		
Liverpool Aintree	46 (39.0%)	46 (37.7%)
Bolton	33 (28.0%)	32 (26.2%)
Wythenshawe Hospital (UHSM)	3 (2.5%)	2 (1.6%)
Macclesfield District Hospital	7 (5.9%)	10 (8.2%)
Pennine Acute Hospitals NHS Trust	29 (24.6%)	32 (26.2%)
Previous psychological therapies for anxiety or depression		
Yes	35 (29.7%)	40 (32.8%)
No	83 (70.3%)	82 (67.2%)
Medication for anxiety		
Yes	15 (12.7%)	13 (10.7%)
No	103 (87.3%)	109 (89.3%)
Medication for depression		
Yes	31 (26.3%)	28 (23.0%)
No	87 (73.7%)	94 (77.0%)
Ethnicity		
White	113 (95.8%)	116 (95.1%)
Other	5 (4.2%)	6 (4.9%)
Employment:		
Economically active	50 (42.4%)	50 (41.0%)
Unemployed	12 (10.2%)	11 (9.0%)
Retired	39 (33.1%)	47 (38.5%)
All other	17 (14.4%)	14 (11.5%)
Education:		
None	22 (18.6%)	22 (18.0%)
School/vocational	69 (58.5%)	70 (57.4%)
Diploma/degree	27 (22.9%)	30 (24.6%)
Civil status:		
In relationship	69 (58.5%)	79 (64.8%)
Separated	27 (22.9%)	22 (18.0%)
Single	22 (18.6%)	20 (16.4%)
Missing	0 (0.0%)	1 (0.8%)
Smoking status		
Never smoked	36 (30.5%)	32 (26.2%)
Ex-smoker	70 (59.3%)	78 (63.9%)
Current smoker	12 (10.2%)	12 (9.8%)
Alcohol units per month	19.0 (31.7)	20.4 (33.5)
Age at first cardiovascular event		
Under 45	17 (14.4%)	18 (14.9%)
45–54	33 (28.0%)	35 (28.9%)
55 and older	68 (57.6%)	68 (56.2%)
Missing	0 (0.0%)	1 (0.8%)
Reason for referral to CR		
Acute coronary syndrome	86 (72.9%)	86 (70.5%)
Following revascularisation	50 (42.4%)	51 (41.8%)
Stable heart failure	18 (15.3%)	15 (12.3%)
Stable angina	3 (2.5%)	6 (4.9%)
Following implantation of defibrillator	3 (2.5%)	3 (2.5%)
Heart valve repair/replacement	4 (3.4%)	12 (9.8%)
Heart transplantation and ventricular assist device	0 (0.0%)	0 (0.0%)
Adult congenital heart disease	1 (0.9%)	0 (0.0%)
Other	1 (0.6%)	0 (0.0%)
Number of previous cardiac events		
None	70 (59.3%)	64 (52.5%)
1	28 (23.7%)	36 (29.5%)
2 or more	20 (17.0%)	22 (18.0%)
Type of previous cardiac events		
Heart attack	20 (17.0%)	22 (18.0%)
Stroke	5 (4.2%)	8 (6.6%)
Pulmonary embolism	2 (1.7%)	2 (1.6%)
Aneurysm	2 (1.7%)	1 (0.8%)
Angina	19 (16.1%)	25 (20.5%)
Arrhythmia	19 (16.1%)	13 (10.7%)
Comorbidities		
Hypertension	66 (55.9%)	63 (51.6%)
Diabetes T1	2 (1.7%)	3 (2.5%)
Diabetes T2	25 (21.2%)	26 (21.3%)
High cholesterol	73 (61.9%)	78 (63.9%)
Peripheral Arterial Disease	5 (4.2%)	5 (4.1%)
Breathing problems or COPD	31 (26.3%)	41 (33.6%)
IBS abdominal problems	11 (9.3%)	20 (16.4%)
Arthritis	47 (39.8%)	51 (41.8%)
Chronic fatigue or fibromyalgia	5 (4.2%)	6 (4.9%)
Multiple sclerosis	2 (1.7%)	0 (0.0%)
Number of comorbidities	3.3 (2.1)	3.5 (2.0)
BMI	31.4 (7.7)	31.1 (7.6)
HADS total score	18.6 (7.8)	17.9 (6.8)
HADS anxiety	10.4 (4.4)	10.3 (4.2)
HADS depression	8.2 (4.4)	7.6 (3.7)
Impact of Event Scale-Revised (IES-R)	32.4 (18.7)	30.9 (18.9)
Meta-cognitions scale 30 (MCQ-30)	61.2 (13.9)	61.7 (15.5)
MCQ-30 negative beliefs subscale	13.4 (4.7)	13.1 (4.6)
EQ-5D-5L Utility scores	0.57 (0.26)	0.60 (0.26)
EQ-5D Visual Analogue Scale	58.7 (19.4)	55.5 (20.8)
CAS-1R	384.0 (190.2)	381.5 (178.6)

Data are mean (SD) or *n* (%).

BMI, body mass index; CAS-1R, Cognitive-Attentional Syndrome 1-Revised; CR, cardiac rehabilitation; HADS, Hospital Anxiety and Depression Scale.

### Randomisation and masking

Patients were randomised via telephone/email link to the Centre for Biostatistics at the University of Manchester. Within each site, participants were stratified by sex and screening HADS score (anxiety score> = 8; depression score> = 8; both> = 8), then allocated to trial arms in a 1:1 ratio using randomised blocks of size 4 and 6. The chief investigator, RAs, and trial statisticians were masked to treatment allocation throughout data collection and analysis. Instances of accidental unmasking were recorded to assess their frequency and severity, which was routinely reviewed by the Trial Steering Committee.

### Interventions

#### Usual CR (Control group)

Usual CR consists of 8 to 10 weeks of group-based exercise classes and educational seminars. Educational seminars focused on health and medical risk factor management. The extent of psychological components varied by site. All sites included educational seminars on stress management and relaxation, which focused on breathing techniques and progressive muscle relaxation. At 3 sites stress management sessions also incorporated 2 cognitive therapy strategies (i.e., challenging negative thoughts and worry decision tree). In addition, 1 site offered a 4-week stress management course as part of CR that included generating and sharing a CBT case formulation based on Greenberger and Padesky [29], mindfulness techniques, and individual counselling with an occupational therapist. Some sites offered additional support to manage psychological distress including a referral for counselling.

#### Home-MCT alongside usual CR (intervention group)

Home-MCT [30] was delivered alongside usual CR. Home-MCT is a self-help manual (hard copy) with 6 modules that participants can complete at their own pace. Modules focus on techniques for reducing worry and rumination and modifying maladaptive metacognitive beliefs that maintain negative repetitive thinking patterns.

Patients also received 3 brief scripted telephone support calls delivered by CR staff who had received basic training in delivering the calls. There were 7 support staff, 3 from Bolton and 4 from Liverpool. The support staff were predominantly CR nurses, with 1 site including a physiotherapist. They completed a workshop delivered by the developer of MCT (AW). Training included didactic teaching, role play, discussion, and studying the treatment manual to facilitate an understanding of the MCT approach and the purpose of the techniques in the manual. In addition, they piloted home-MCT support calls with 5 volunteers each (total of 1.5 hours of practise), after which an additional 1-day workshop was delivered, which focused on enhancing support skills.

The first scripted call was 10 to 15 minutes and introduced the structure of the manual and helped users think of a timetable for completing the modules. The second and third calls (approximately 30 minutes each) reviewed the modules completed to date and helped participants consolidate what they had learned from the exercises, encouraging widening of practise of techniques, reviewing unhelpful coping behaviours, and increasing motivation to change them. The support calls did not consist of actively delivering MCT intervention techniques (e.g., practising exercises) but patients were asked questions to help them reflect on changing beliefs about worrying (e.g., “Do you believe worrying is uncontrollable?”) and changing behaviours.

#### Data collection

**Fig 1:**
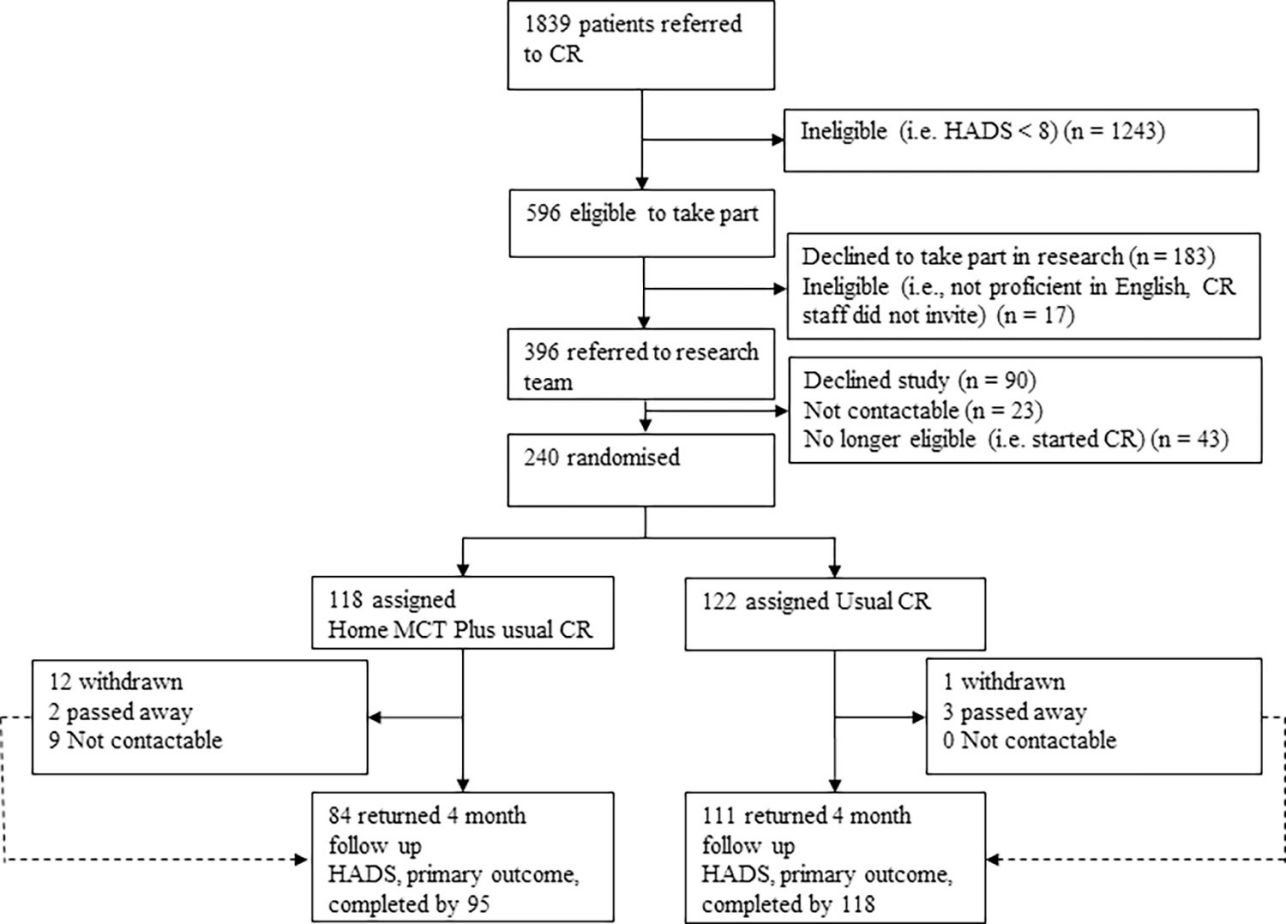
CONSORT Flowchart. CR, cardiac rehabilitation; Home-MCT, home-based metacognitive therapy.

As depicted in Fig 1, the measurement time points included baseline (pre-CR) and 4-month follow-up (posttreatment). Baseline assessments were completed face-to-face with an RA and a range of options were offered for completing follow-up assessments: by post, face-to-face with an RA at the patient’s home or NHS centre, or over the telephone. Patients received £5 cash for completing the baseline assessment and received a £10 shopping voucher for each follow-up assessment returned. All outcome measures detailed below were collected at each time point.

### Outcomes

The primary outcome was the HADS [27] total score at 4 months. Separate HADS anxiety and depression subscales analysis were secondary outcomes. Scores on the HADS for each subscale range from 0 to 21. The HADS has 4 cutoffs that have been used: scores from 0 to 7 are categorised as normal, scores from 8 to 10 are mild, scores from 11 to 14 are moderate, and scores from 15 to 21 are severe [28].

Four other measures were used to evaluate the secondary outcomes. The Impact of Event Scale–Revised (IES-R) [31] was used to assess symptoms of post-traumatic stress. Total scores on the IES-R range from 0 to 88 and includes 3 subscales: Intrusions, Avoidance, and Hyperarousal. Scores of 24 to 32 indicate probable post-traumatic stress disorder is a clinical concern, whereas scores of 33 to 88 indicate a likely diagnosis of post-traumatic stress disorder [31]. The Metacognitions Questionnaire 30 (MCQ-30) [32] was used to assess metacognitive beliefs (an underlying psychological causal variable) plus the “negative beliefs about thoughts concerning uncontrollability and danger” subscale of the MCQ-30 to represent the primary mechanism targeted in MCT; other MCQ-30 subscales were omitted to reduce multiple testing. This questionnaire yields a total score ranging from 30 to 120, where higher scores indicate greater maladaptive metacognitive beliefs. The EQ-5D-5L [33] assessed quality of life (EuroQol 5 dimensions 5 levels; both the utility score and visual analogue scale [VAS] score) with scores derived using the recommended methods of the National Institute for Health and Care Excellence [34]. The Cognitive-Attentional Syndrome 1–Revised (CAS-1R) [35], assessed measure repetitive negative thinking and unhelpful coping behaviours, considered as mechanism variables. The CAS-1R includes 10-items assessing the degree to which individuals have been worrying and/or focusing attention on threats, the degree to which they hold negative metacognitive beliefs, and positive metacognitive beliefs about worry. Each CAS-1R item is scored on a scale from 0 to 100 with higher scores indicating greater unhelpful metacognitive strategies or greater conviction in unhelpful metacognitive beliefs. All outcome measures were completed at each time point with an RA blind to allocation.

Adverse events were monitored throughout by healthcare professionals delivering CR and reported and assessed as related or unrelated to the study. Adverse and serious adverse events were reviewed on a quarterly basis at the study executive committee meetings.

### Statistical analysis

The trial was designed to detect a standardised mean difference (SMD) between trial arms of 0.4 in HADS total score at 4-months follow-up with 90% power, where 0.4 is in the middle of the range of effect sizes reported for other forms of psychological interventions for depression [36]. We assumed a 0.5 correlation between HADS at baseline and 4-month follow-up and 20% attrition. This indicated a total recruitment target of 246 (123 per arm). In March 2020, the United Kingdom was put into lockdown as a result of the Coronavirus Disease 2019 (COVID-19) pandemic. COVID interfered with participation in CR and also with the entry of CR attendance data into the database. Since it was unknown how long the lockdown would be in place, a decision was taken to review the data collected to date, to determine if it was feasible to halt any further data collection. On the basis of the number recruited to date (n=240) and a lower than expected 4-month attrition rate on the primary outcome and in discussion with the TSC, it was decided that the study was sufficiently powered without any further recruitment.

Analysis was conducted in accordance with a prespecified analysis plan detailing the analytical models, primary and secondary outcomes, choice of covariates, sensitivity analyses, and all other key aspects of the analysis. The plan was finalised and approved by the Trial Steering Committee prior to data analysis or unmasking. The primary analyses used intention-to-treat principles. For each of the continuous outcomes, a linear mixed effects regression model was applied incorporating both time points (baseline, 4 months). Prespecified covariates in the model were the design stratification actors of hospital site and sex, plus age and medication for depression or anxiety (never taken/currently taking/taken in the past). All other potential covariates (Table 1) were below predefined imbalance criteria for sensitivity testing (SMD >0.25 or category difference of >10% between arms). Patient was a random effect in the model and we assumed an unstructured covariance matrix. The effects of the intervention at 4-month follow-up were examined using the treatment group by time point interaction term from the model. No adjustments for multiple testing were applied and an alpha value of 5% was used throughout. We ran sensitivity analysis using MI to assess robustness of results against missing values. There were no missing outcome scores at baseline and only 4 missing covariate values; therefore, these were imputed by simple regression imputation using all available variables at baseline but excluding treatment arm. MI was then used to impute missing outcome values at 4 months, using the full set of variables and including the interaction term between treatment arm and time point (for consistency with the analysis model). We used the chained- equations MI procedure and 20 MI datasets. One outcome measure, the EQ-5D utility score, had a skewness exceeding the threshold of 1.0 specified in our analysis plan and so the *p*-values for this outcome were validated in a sensitivity analysis using bootstrapped standard errors based on 10,000 repetitions.

To aid interpretation, we computed effect sizes in the form of SMDs between groups. For consistency with Cochrane reviews and to enable cross-study comparisons, these were computed as the adjusted mean difference between groups from the mixed effects model divided by the pooled standard deviation of change scores from baseline [37]. A general rule of thumb for interpreting the SMD is that 0.2 represents a small effect, 0.5 a medium effect, and 0.8 a large effect [38]. To facilitate interpretation of the clinical importance of findings, we also computed the reliable change index (RCI [39]) for the HADS total score at 4-month follow-up. The RCI represents the difference between 2 measurements made on a single individual that would be statistically significant at *p* < 0.05. A Cronbach alpha of 0.91 derived from the control sample at 4 months was used as the estimate of reliability for a usual CR population, since baseline HADS scores had restricted variance due to being a study eligibility criterion. We calculated that a reduction of 7 points in an individual’s score represented statistically reliable improvement, while an increase of 7 points represented reliable deterioration in symptoms.

Being based on statistically significant change within an individual, the RCI can be conservative. We therefore conducted an equivalent analysis based on a published minimal clinically important difference (MCID) for the HADS anxiety and depression subscales of 2 points [40]. To operationalise this for our primary outcome of the HADS total, we used an MCID of 3 points, requiring at least 1 subscale to change by 2 points or more.

We observed a higher attrition rate by 4-months follow-up in the MCT+CR arm. Sensitivity analysis based on MI assumes data is missing at random, which is unlikely in this case. We therefore conducted 2 additional sensitivity analyses to account for differential attrition. The first assumed that patients who dropped-out experienced no change in their outcome scores between baseline and 4 months (a last observation carried forward (LOCF) analysis). The second sensitivity computed the mean 4-month score required among drop-outs to reduce the treatment effect between arms to zero, for each outcome. This score was expressed as a percentile of the outcome’s score distribution among controls at 4 months to aid interpretation. Analyses were conducted using Stata version 14.

## Results

A total of 1,839 patients were referred to CR between April 2017 and March 2020, of which 596 had an elevated HADS score and were screened for eligibility (see Fig 1). Approximately 17 patients did not meet full eligibility criteria (e.g., not proficient in English), resulting in 396 eligible patients. Of these, 90 declined to take part, 23 were uncontactable, 43 no longer met the inclusion criteria (e.g., began taking antidepressants, presence of suicidality, experiencing symptoms of psychosis, started CR). Consequently, 240 patients were consented to the trial of which 118 (49.2%) were randomly allocated to Home-MCT plus CR and 122 (50.8%) to CR alone. Mortality rates were similar in the 2 arms (2 versus 3 individuals) but other forms of attrition were higher under the MCT+CR condition, where 21 participants (18%) withdrew or were uncontactable at 4 months, compared to just 1 individual (1%) under CR (Fisher’s exact test: *p* < 0.001). There were 8 incidents of unmasking, which involved RAs accidently being unblinded to patient allocation. There were no cases of the trial statisticians or chief investigator being unmasked.

Table 1 provides demographic and clinical data for the sample at baseline. The groups were well balanced on all measured variables. Approximately 37% of patients in the MCT+CR arm were female compared to 42% of patients in the CR arm, mean ages were 60 and 61 years and exposure to previous psychological therapy 30% and 33%, respectively. Mean HADS total scores were very similar (18.6 versus 17.9), as were MCQ30 (61.2 versus 61.7), negative beliefs subscale (13.4 versus 13.1), CAS-1R (384.0.0 versus 381.5), and EQ-5D-5L utility scores (0.57 versus 0.60). Differences were only a little larger with regard to EQ-5D VAS (58.7 versus 55.5) and IES-R (32.4 versus 30.9). The mean intervals between baseline and 4-month assessments were similar for both arms (MCT+CR: 133.8 days; CR: 132.2 days).

**Fig 2:**
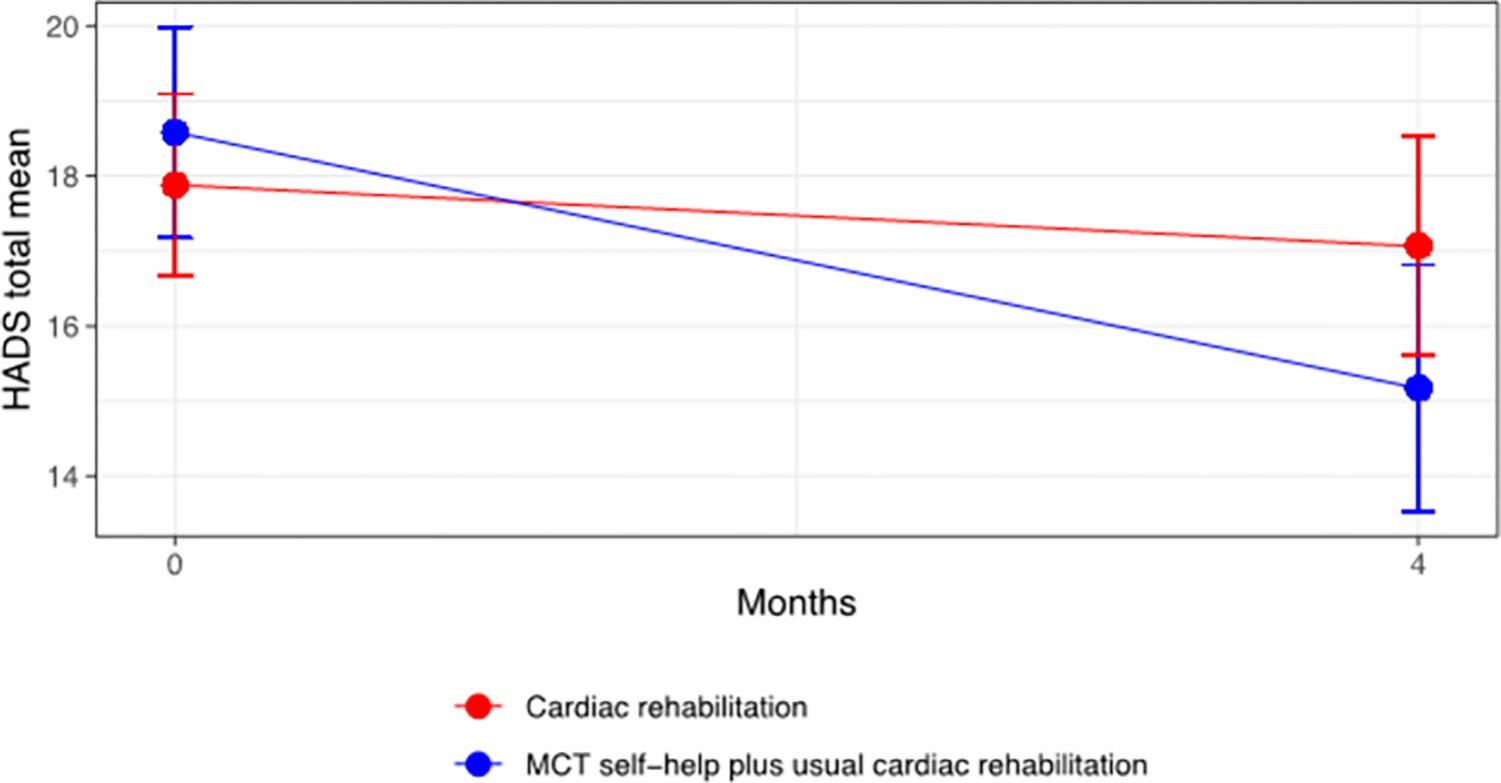
Mean HADS total scores and 95% confidence intervals for each trial arm at each assessment point, for complete cases only. HADS, Hospital Anxiety and Depression Scale.

Results of the main analysis of primary and secondary endpoints are summarised in Table 2. The adjusted group difference on the primary outcome (HADS total score at 4 months) significantly favoured the MCT+CR arm (adjusted mean difference = −2.64 [−4.49 to −0.78], *p* = 0.005, SMD = 0.38). Fig 2 presents mean HADS total scores and 95% confidence intervals for each trial arm at each assessment point, for complete cases only. The CR group mean score demonstrated little change, in comparison with a notable reduction in the mean for the Home-MCT+CR group.

**Table 2:** Summary of analyses of primary and secondary outcomes.

	Home-MCT plus CR		CR alone		Adjusted difference		Adjusted difference using multiple imputation		Effect size* (SMD)	Sensitivity analysis: LOCF	Sensitivity analysis: mean (percentile) 4-month score required by drop-outs to reduce group difference to zero.
	n**	4-month mean (SD)	n**	4-month mean (SD)	Mean (95% CI)	*p*-value	Mean (95% CI)	*p*-value		*p*-value	
HADS total	95	15.17 (8.20)	118	17.06 (8.05)	−2.64 (−4.49 to −0.78)	0.005	−2.45(−4.43 to −0.47)	0.015	0.38 (0.11 to 0.64)	0.018	30 (92.4%)
HADS anxiety	95	8.42 (4.54)	118	9.45 (4.51)	−1.18 (−2.26 to −0.10)	0.032	−1.10 (−2.27 to −0.06)	0.063	0.29 (0.02 to 0.55)	0.103	15 (85.6%)
HADS depression	95	6.75 (4.51)	118	7.61 (4.30)	−1.46 (−2.48 to −0.45)	0.005	−1.35 (−2.45 to −0.25)	0.016	0.39 (0.12 to 0.66)	0.009	15 (94.1%)
IES-R	83	21.52 (18.15)	110	28.14 (21.29)	−8.50 (−13.22 to −3.79)	<0.001	−8.08 (−13.24 to −2.93)	0.002	0.51 (0.23 to 0.80)	0.005	58 (89.1%)
MCQ-30 total score	83	55.19 (13.90)	109	62.60 (18.62)	−6.74 (−10.53 to −2.94)	<0.001	−7.10 (−11.25 to −2.95	0.001	0.50 (0.22 to 0.78)	0.002	86 (88.1%)
MCQ-30 negative beliefs subscale	84	10.92 (4.26)	109	13.37 (4.95)	−2.80 (−4.07 to −1.53)	<0.001	−2.77 (−4.11 to −1.44)	<0.001	0.61 (0.34 to 0.89)	<0.001	25 (100%)^^^
EQ5D Utility score	83	0.63 (0.30)	110	0.59 (0.28)	0.06 (<-0.01 to 0.13)	0.054	0.05 (−0.02 to 0.12)	0.153	0.26 (−0.01 to 0.57)	0.086	0.33 (17.3%)
EQ5D visual analogue scale	84	65.51 (18.95)	109	61.65 (20.43)	0.48 (−5.45 to 6.42)	0.874	0.65(−5.58 to 3.01)	0.836	0.02 (−0.25 to 0.30)	0.754	65 (46.8%)
CAS	84	273.21 (158.69)	110	353.00 (215.45)	−85.99 (−138.05 to −33.92)	0.001	−83.85 (−137.87 to −29.83)	0.002	0.46 (0.18 to 0.73)	0.012	650 (81.5%)

*Adjusted mean difference divided by pooled standard deviation of change scores from baseline.

**Numbers of patients with useable scores varied by outcome.

^^^The score required is higher than the maximum possible for the scale, 24.

CI, confidence interval; CR, cardiac rehabilitation; HADS, Hospital Anxiety and Depression Scale; IES-R, Impact of Event Scale-Revised; LOCF, last observation carried forward; SD, standard deviation.

Patients in the MCT+CR arm achieved significantly lower mean HADS anxiety subscale score (−1.18 [−2.26 to −0.10], *p* = 0.032, SMD = 0.29), plus a lower HADS depression subscale mean score (−1.46 [−2.48 to −0.45], *p* = 0.005, SMD = 0.39). Most other secondary outcomes also favoured the MCT intervention: IES-R (−8.50 [−13.22 to −3.79], *p* < 0.001, SMD = 0.51); MCQ-30 total scores (−6.74 [−10.53 to −2.94], *p* < 0.001, SMD = 0.50); negative beliefs subscale scores (−2.80 [−4.07 to −1.53], *p* < 0.001, SMD = 0.61) and the CAS-1R (−85.99 [−138.05 to −33.92], *p* = 0.001, SMD = 0.46). EQ-5D utility scores demonstrated no statistically significant difference (0.06 [<-0.01 to 0.13], *p* = 0.054, SMD = 0.26); similarly, the difference on the EQ-5D-VAS was not significant (0.48 [−5.45 to 6.42], *p* = 0.874, SMD = 0.02). Sensitivity analysis using MI changed only the result for HADS anxiety which ceased to be statistically significant. The use of a bootstrapped standard error did not alter the result for the skewed utility outcome.

Investigation of the impact of differential attrition on the findings using LOCF resulted in no changes in statistical significance for the primary outcome and most secondary outcomes. However, the group difference on the HADS anxiety subscale became nonsignificant (Table 2). To fully eliminate any group difference on the primary outcome, drop-outs would require a mean HADS total follow-up score of 30 points or more, which is higher than the scores posted by 92% of control participants. For all other outcomes except the EQ-5D, the mean score would need to exceed the score posted by 80% of controls.

**Table 3:** Patients with a reliable change in HADS total score by 4 months by treatment group, using complete cases.

Reliable change	Home-MCT plus CR	CR alone	Total
**No change**	52 (54.7%)	66 (55.9%)	118 (55.4%)
Improved*	34 (35.8%)	30 (25.4%)	64 (30.1%)
Deteriorated**	9 (9.5%)	22 (18.6%)	31 (14.6%)

*HADS total score reduced by 7 points or more from baseline.

**HADS total score increased by 7 points or more from baseline. Note: “No reliable change” refers to not reaching the threshold of changing by at least 7 points; it does not imply no change at all.

HADS, Hospital Anxiety and Depression Scale.

**Fig 3:**
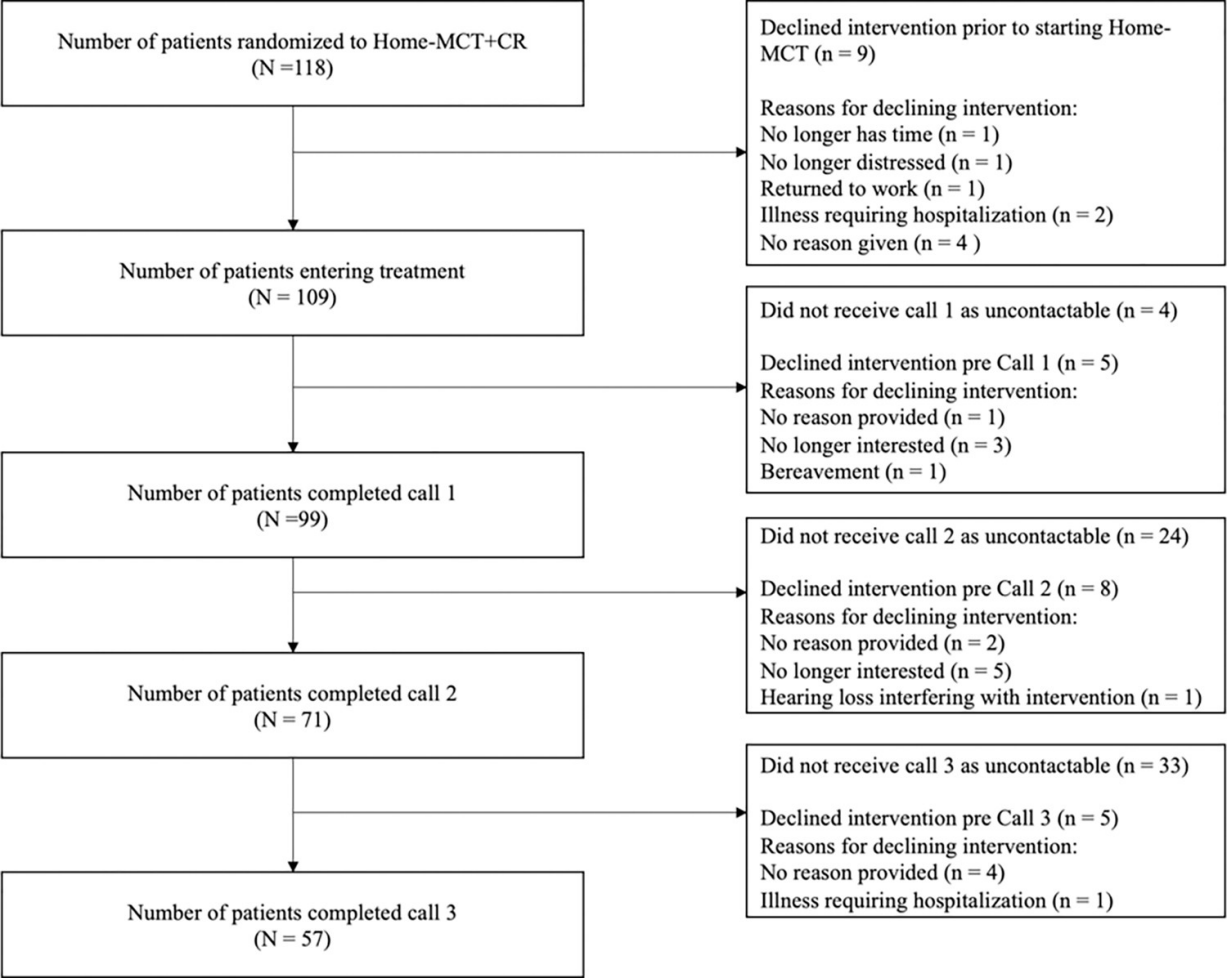
Home-MCT patient flow. CR, cardiac rehabilitation; Home-MCT, home-based metacognitive therapy.

Patients in both trial arms attended routine CR sessions at their site as members of larger groups (of up to 15) including patients with CVD not part of the trial. Among patients returning the 4-month follow-up questionnaire, attendance at CR exercise sessions was high in both groups, with a median of 5 (IQR 2 to 6) out of 10 sessions attended by CR arm patients and 5 (IQR 2 to 7) by MCT+CR patients, a nonsignificant difference (*p* = 0.951). Attendance at CR educational sessions was lower (medians: CR 4 (IQR 1 to 6), MCT+CR 4 (IQR 0 to 6)) and did not differ between groups (*p* = 0.657). Approximately 76% of participants in the Home-MCT arm entering treatment completed 4 or more Home-MCT modules, our criteria for a clinically effective exposure [21]. For further details on participant completion of home-MCT, see Fig 3. As both conditions involved treatment (and case management) as usual, this meant that additional psychological treatment could be sought. Across the follow-up period, 1 patient under MCT+CR and 6 under CR received new psychological therapy outside of CR or MCT; the difference was not statistically significant (Fisher’s exact test: *p* = 0.139); however, the numbers involved were small.

### Strength and clinical significance of treatment effects

The percentage of patients reaching threshold for a reliable improvement on HADS total was 25% in CR compared to 36% in Home-MCT+CR, the percentages exhibiting reliable psychological deterioration were 19% (approximately 1 in 5 patients) in CR compared to 10% (1 in 10 patients) in MCT+CR (Table 3). Based on the MCID, clinical improvement was 36% in CR compared to 59% in Home-MCT+CR, while the percentages exhibiting psychological deterioration were 31% in CR compared to 16% in MCT+CR (Table 4).

**Table 4:** Patients with a minimal clinically important change in HADS total score by 4 months by treatment group, using complete cases.

Minimal clinically important change	Home-MCT plus CR	CR alone	Total
**No change**	24 (25.3%)	39 (33.1%)	63 (29.6%)
Improved*	56 (59.0%)	43 (36.4%)	99 (46.5%)
Deteriorated**	15 (15.8%)	36 (30.5%)	51 (23.9%)

*HADS total score reduced by 3 points or more from baseline.

**HADS total score increased by 3 points or more from baseline. Note: “No reliable change” refers to not reaching the threshold of changing by at least 3 points; it does not imply no change at all.

HADS, Hospital Anxiety and Depression Scale.

### Adverse events reporting

Safety and adverse events (increased suicidality, death, self-injury) associated with the treatments were monitored throughout the trial. No adverse events were reported.

## Discussion

This trial showed that the addition of a home-based self-help MCT significantly reduced overall anxiety and depression symptoms (total HADS) in CVD patients undergoing cardiac rehabilitation. Significantly greater improvement was observed in the primary outcome, HADS total at 4-months and in most secondary outcomes. Post-traumatic stress symptoms (secondary psychological outcome) appeared to respond particularly well in the self-help MCT condition. These outcomes showed for the first time that self-help MCT was an effective intervention when added to CR, across anxiety, depression, and trauma symptoms in patients with CVD.

An analysis of separate HADS subscales indicated greater improvements in both anxiety and depression symptoms in the MCT group. However, it should be noted that the treatment effect on anxiety was smaller and not significant under MI and LOCF sensitivity analyses accounting for attrition. All other significant effects were robust against sensitivity analyses.

The HADS effect sizes were lower than those obtained in our previously published face-to-face group based MCT trial of CR patients [23]. The current effects sizes of 0.38 (HADS total), 0.29 (HADS anxiety), 0.39 (HADS depression) compare with the 4-month effects in our previous therapist delivered treatment trial of 0.52 on HADS total (0.44 anxiety and 0.47 depression). These results suggest that there may be a reduction in effectiveness when using self-help MCT (especially on anxiety) compared with therapist delivered MCT. However, the results for PTSD symptoms appear to give a different perspective, where the current effect of 0.51 is higher than the 0.28 obtained in the group intervention, despite the fact that the samples in each study had similar baseline scores. An implication that should be explored in future is that self-help MCT may be particularly effective in treating PTSD symptoms in CR patients.

Compared to treatment as usual, the home-based treatment had a significantly greater effect on underlying psychological mechanisms considered to contribute to excessive and chronic anxiety and depression symptoms. We found greater improvements in worry, rumination, and unhelpful coping behaviours, as well as greater reductions in maladaptive metacognitive beliefs (total MCQ score and uncontrollability and danger subscale). It is notable that while the controlled effect for metacognitive beliefs is only slightly smaller in this study compared to our earlier group-MCT intervention study, the effect on the cognitive attentional syndrome (CAS) measure is seemingly lower here. In the present study, the SMD for CAS was 0.43 and this compares with an SMD of 0.73 in our earlier therapist delivered group intervention. The reduced impact on causal mechanisms (CAS, i.e., worry, rumination, unhelpful coping) might account for the reduced effectiveness of self-help MCT on HADS compared with the effects observed in our earlier study of face-to-face group MCT.

Twenty-four percent of participants returning the end of treatment questionnaire completed less than 4 Home-MCT modules and attrition from the study was significantly higher in the MCT+CR arm. Hence, it appears that Home-MCT did not appeal to some patients. Nevertheless, the completion rates of Home-MCT compare favourably to previous studies of psychological guided self-help interventions. For example, Lundgren and colleagues [41] evaluated guided web-based CBT for heart failure therapy with depression (*n* = 50). They found that 60% of patients completed 4 out of the 7 modules, with only 24% completing all 7 modules.

The results we obtained compare favourably with the results of other studies that have used a range of treatment techniques. In a study similar to the present study, Lewin and colleagues [16] examined the effects of the “Heart Manual,” a self-help intervention in post MI rehabilitation. The intervention improved HADS anxiety compared to usual care but the between-group effects on depression were not significant across time. Moving beyond only self-help, meta-analyses of 35 RCTs of people with CVD treated for anxiety and depression using a variety of psychological interventions when compared with usual care, show effect sizes of 0.24 (95% CI, 0.09 to 0.38) for anxiety and 0.27 (95% CI, 0.15 to 0.39) for depression [42]. Our present results suggest effects for home-based MCT that might be comparable in anxiety, although this must remain tentative as significance was lost under sensitivity analysis, while our results for depression appear stronger than previous studies aggregated across intervention types. It is important to note that the effects of MCT in the present study and in our earlier group therapist-delivered trial in CR are smaller than the effect sizes found in trials of MCT within mental health services. It is likely that this is due to ongoing background distress that normally accompanies the uncertainty, life-limiting, and disabling effects of long-term medical conditions. However, it must be borne in mind that the design of the current study is such that we are testing the additional benefit of MCT when added to existing CR which itself contained various psychological interventions. Thus, the controlled effect size observed here is not readily comparable with effect sizes seen in trials of non-overlapping treatments.

We failed to find a significant effect on EQ-5D utility scores, although the *p*-value approached significance.

### Strengths

The strengths of the current study include the recruitment of a large sample size, clearly specified a priori outcomes and analysis plan, recruitment from multiple treatment sites, and blinding of investigators. We used an evidence-based model and treatment on which to base the self-help intervention.

### Limitations

The main limitation is the absence of longer term (e.g., 12-month) follow-up data. This study was an extension of a pilot feasibility study of self-help MCT that was approved under variation to contract and we did not have the time and resource to collect the longer term follow-up data. We cannot determine the effects of MCT alone in this study as it was designed to address a question of the “added effect” of self-help MCT when offered alongside CR. It is possible that combining MCT with psychological techniques included in some CR packages may impair rather than enhance MCT effects, because they rely on opposing mental processes [43].

## Conclusion

Self-help metacognitive therapy significantly improved total anxiety and depression symptoms when added to usual cardiac rehabilitation. The treatment also resulted in significantly improved traumatic stress symptoms and impacted on hypothesised psychological causal variables. An important incidental finding was preliminary evidence that the addition of self-help MCT might also reduce rates of psychological deterioration in CVD patients. The implication is that self-help MCT could be offered to distressed CVD patients as an effective psychological intervention in addition to or as an alternative to group-based therapist-led MCT.

## Supporting information

S1 Statistical Analysis Plan PATHWAY study: Statistical Analysis Plan for WS3+ RCT outcomes at 4 months. (DOCX)

## Abbreviations

BACPR: British Association for Cardiovascular Prevention and Rehabilitation
CAS: cognitive attentional syndrome
CBT: cognitive behavioural therapy
COVID-19: Coronavirus Disease 2019
CR: cardiac rehabilitation
CVD: cardiovascular disease
HADS: Hospital Anxiety and Depression Scale
iCBT: internet CBT
IES-R: Impact of Event Scale–Revised
LOCF: last observation carried forward
MCID: minimal clinically important difference
MCT: metacognitive therapy
MI: multiple imputation
NHS: National Health Service
RA: research assistant
RCI: reliable change index
RCT: randomised controlled trial
SMD: standardised mean difference
VAS: visual analogue scale
VTC: variation to contract
