# Supplementary material for: Metacognitive therapy home-based self-help for anxiety and depression in cardiovascular disease patients in the UK: A single-blind randomised controlled trial
Source: PLoS Med. 2023 Jan 31;20(1):e1004161. doi: 10.1371/journal.pmed.1004161 (PMC9888717; doi:10.1371/journal.pmed.1004161)
Supplement: S1 Statistical Analysis Plan — (DOCX) [file pmed.1004161.s001.docx]

**PATHWAY study: Statistical Analysis Plan for WS3+ RCT outcomes at 4 months**

**Version 2 with amendment to the MCQ-30 secondary outcome specification 24/03/2021**

1. **Introduction**

This document presents the SAP for WS3+, an extension of the WS3 study. The original Pathway WS3 was designed as a feasibility study of home-based MCT. The study included quantitative and qualitative work to assess the feasibility of conducting an RCT of Home-MCT, including acceptability to patients and the processes of recruitment, randomisation, treatment, and data collection at baseline and follow-up; together with a pilot study designed as a miniature version of a full RCT to test whether all the components of the main study would work together smoothly. Additionally, to collect data on patient variables and outcome measures to inform the design and sample size for a full-scale trial and establish provisional evidence of the effectiveness of Home-MCT at reducing anxiety and depression symptoms.

In January 2019 analysis of recruitment and qualitative data collected under the study concluded that provision of Home-MCT had proved acceptable to patients, recruitment had been to target and on time, and that a high 4-month retention rate had been achieved. Analysis of the pilot baseline outcome data indicated that rates of missing data were low and the outcome measures demonstrated sufficient variability to detect change. With a view to potential extension of the data collection (see below), we used the following strategy: 1) all analysis was conducted without breaking the blinding of the PI or trial statisticians; 2) no separate trial-arm analysis was undertaken, and 3) none of the 4- or 12-month outcome data was examined, except for 4-month completion rates. Consequently, no estimate of the effectiveness of Home-MCT was made from the data collected under the pilot since our aim was to extend data collection and use the pilot as an internal-pilot study.

On the basis of these results and after full discussion with the trial steering committee (TSC), a variation to contract (VTC) application was made to the NIHR to utilise a project underspend to extend the study into a definitive trial of the intervention, called WS3+, through the recruitment of additional patients. No changes were required to the method of recruitment into the trial and the randomisation procedure did not change. Also, all outcome measures remained the same, including total score on the HADS as the primary outcome. In view of there being no substantial changes to any of the trial processes or instruments and that the pilot data had not been unmasked or any arm-based analysis conducted, the VTC proposed that the pilot trial sample and the additional participants be combined into a single sample for the purposes of analysis of the WS3+ RCT.

NIHR approval for the variation to contract was obtained on 12/03/2019. Recruitment to WS3+ commenced on 27/05/2019, representing a 65 week break since recruitment of the last participant to WS3. Two differences from WS3 should be noted: (1) in order to achieve the target sample, recruitment was extended to include two additional hospital Trusts; (2) to complete WS3+ within the timeline of the Pathway programme, follow-up data collection was restricted to 4 months HADS (the primary outcome) and no 12-month follow-ups were conducted.

**Sample size**

The WS3 pilot trial had a planned total sample size of 108, using 1:1 random assignment to Home-MCT plus usual CR or usual CR alone. Participant allocation was undertaken by a blinded statistician using a sequence based on randomised blocks of size 6 and 4, stratified by site, gender and whether they suffered from anxiety, depression or both.

WS3+ utilised an identical randomisation procedure and was powered to detect an effect size of 0.4 on total HADS score at 90% power, assuming a 0.5 correlation between HADS at baseline and FU 20% drop-out at 4 months follow-up (based on WS2). This indicated a total recruitment target (i.e. including the internal pilot participants) of 123 per arm, aiming to collect 4month FU on 98 of these in each arm.

In March 2020 the UK was put into lockdown as a result of the covid-19 pandemic. Covid interfered with participation in CR and also with the entry of CR attendance data into the database. Since it was unknown how long the lockdown would be in place, a decision was taken to review the data collected to date, to determine if it was feasible to halt any further data collection. The table below shows the target and achieved samples at baseline and 4 months as of 06/04/2020

WS3+ (Total Target: 246):

|  | MCT + CR | | | CR | | |
| --- | --- | --- | --- | --- | --- | --- |
|  | Target sample | Achieved sample | Number known to have completed CR | Target sample | Achieved sample | Number known to have completed CR |
| Baseline | 123 | 119 | - | 123 | 121 | - |
| 4 months FU | 98 | 76 (84)* | 58 | 98 | 100 (105)* | 61 |
| *including patients who returned the primary outcome (HADS) only | | | | | | |

On the basis of the samples for which the primary outcome at 4-months was available (MCT+CR=84; CR=105), the available power to detect the target effect of 0.4 was computed to be 88%. For the secondary outcomes (MCT+CR=76; CR=100) the power to detect an effect of 0.4 was 86%.

On the basis of the above, in discussion with the TSC it was decided that recruiting additional participants into WS3+ would have quite limited benefit, and that study resources would be better deployed at aiming to collect the outstanding 4 month FUs from current participants. This should also aim to reduce the imbalance between trial arms in the numbers with outstanding 4-month FUs to collect.

1. **Main analyses of Primary Outcomes**

*Research Null hypothesis:*

There is no significant difference in the primary outcome between usual cardiac rehabilitation (CR) and home-based (self-help) metacognitive therapy (MCT) plus usual cardiac rehabilitation at 4 months. The primary endpoint will be the following outcome measured at 4 months:

| **Primary outcome** | **Description** |
| --- | --- |
| Hospital Anxiety and Depression Scale (Total score) | A 14-item measure of anxiety and depression designed for use in non-psychiatric hospital clinics. |

**Planned statistical methods**

Test of the intervention: The effect of the intervention will be examined using the treatment group by time-point interaction term from a multi-level, mixed effects, regression model analysis combining both time-points (baseline and 4 months). The primary endpoint is HADS total score at 4 months, for which the interaction component relating to 4 months (relative to baseline) will be used.

The primary analysis will take the form of an intention-to-treat analysis using complete cases. This will be supplemented by a sensitivity analysis using multiple imputation, allowing us to assess the robustness of the results against missing values (see below). Other sensitivity analyses will be conducted as appropriate (see below). Analysis will be conducted using Stata 15 and an alpha level of 5%.

**Analysis method**

The primary outcome is assumed to be a continuous longitudinal variable for which linear regression is appropriate. The model will have two levels: time-points within patients**.** Random effects will be applied to the patient-specific intercepts.

1. Outcome: HADS total score at baseline and 4 months.
2. Fixed effects: trial arm, time-point, arm x time-point, design variables (hospital site, sex), other pre-specified covariates and additional covariates (see Choice of Covariates, below).
3. Random effects: patient.

Time-point will be treated as a discrete variable.

In terms of the covariance matrix chosen for the mixed effects model, both an unstructured and a first order autoregressive covariance matrix will be explored. The one with the lowest Bayesian information criteria will be selected.

Alternative tests if violation of distributional assumptions

See distributional tests below.

Sensitivity analysis

See relevant section below

1. **Analyses of Secondary Outcomes**

Research hypotheses:

There are no significant differences in the secondary outcomes between usual cardiac rehabilitation (CR) and home-based (self-help) metacognitive therapy (MCT) plus usual cardiac rehabilitation at 4 months. For the secondary outcomes, tests of an intervention effect will be assessed using the mixed-effects model interaction between treatment arm and time-point.

| **Secondary outcome** | **Description** |
| --- | --- |
| HADS anxiety score | Score based on summing across the 7 anxiety-specific items from the HADS |
| HADS Depression score | Score based on summing across the 7 depression-specific items from the HADS |
| Metacognitions Questionnaire 30 (MCQ-30), the 5 sub-scale scores | MCQ30 is a 30 item measure of beliefs, responses rated on a 4-point Likert scale. Each of the 5 subscales will be analysed separately – the MCQ30 total score will not be analysed.  **Amendment 24/03/2021**  The above text has been found to have been incorrectly retained from an early draft of the SAP and does not express the final planned analysis of the MCQ-30. The below amendment is made prior to any data unblinding.  The trial will analyse the MCQ-30 total score and the ‘negative beliefs about thoughts concerning uncontrollability and danger’ subscale, in line with the analysis of the Pathway WS2 RCT. In previous work we found the MCQ-30 to have a bifactor structure, consisting of a dominant general factor alongside the five sub-scales. The total score has been selected as an outcome to represent the general factor, and the uncontrollability and danger subscale has been selected  as reflecting the primary mechanism targeted in MCT. The remaining sub-scales are omitted to reduce multiple testing. |
| Cognitive Attentional Syndrome-1r (CAS-1r), total score | A 16 item measure assessing individuals’ metacognitive strategies and knowledge. Scored on a scale from 0 to 100, with higher scores indicating more use of metacognitive strategies or greater conviction in metacognitive beliefs. |
| Impact of Event Scale-Revised | A 22-item measure of trauma-related symptoms associated with specific life events. Participants rate how distressing each ‘difficulty’ has been over the past week with respect to their ‘heart event which occurred recently’. Each item is on a five-point scale ranging from 0 (‘not at all’) to 4 (‘extremely’). The IES-R yields a total score ranging from 0 to 88. |
| EQ-5D-5L | A standardised measure of health-related quality of life across five dimensions: mobility, self-care, usual activities, pain/discomfort and anxiety/depression. Each dimension has five response categories ranging in severity from “no problems” to “extreme problems”.  We will analyse the utility scores derived from the instrument. |
| EQ-5D VAS | Score on the general health Visual Analogue Scale of the EQ-5D |

In addition to the above, further patient data will be collected using the Economic Patient Questionnaire (EPQ), designed to collect data on outpatient services and non-hospital-based health and social care use. The EPQ assesses three areas, namely use of primary and community-based health services, social support services accessed outside the hospital, and aids and equipment used as part of care. The EPQ will be used in the health economics analysis and as such will be covered by the HE Analysis Plan and is not discussed further in the current document.

**Planned statistical methods**

Analysis of secondary outcomes:

All of the secondary outcomes will be treated as longitudinal continuous variables. For each secondary outcome, the effect of the intervention will be examined using the 4-month trial arm by time-point interaction term from the mixed effects model. The model will have two levels: time-points within patients. Random effects will be applied to the patient-specific intercepts.

- Fixed effects: trial arm, time-point, arm x time-point, design variables (hospital site, sex), other pre-specified covariates and additional covariates (see Choice of Covariates, below).
- Random effects: patient.

Sensitivity analysis

See relevant section below

Alternative tests if violation of distributional assumptions

See distributional tests below.

1. **Sensitivity analysis**

All of the above primary and secondary analyses will be supplemented with at least one sensitivity analysis where appropriate.

Imputation methods will be used to impute missing values at both time points and the main analysis re-run using this data. Imputation will be done in two stages: first, missing baseline values will be imputed using all available variables at baseline, but excluding treatment arm; second, missing values at 4 months follow-up will be imputed, using the full set of variables and including the interaction term between treatment arm and time-point (for consistency with the analysis model). If missing data at baseline is under 5% these will be imputed with simple regression imputation, otherwise multiple imputation. Follow-up values will utilise multiple imputation.

The extent of the missing data will determine how many MI datasets will be generated.

Additional sensitivity analyses will be conducted where the outcome variable does not match the distributional assumptions of the model, and/or if trial-arm imbalance on multiple covariates is present.

1. **Distributional tests**

The distribution of each outcome variable will be examined. If for any of these, their measure of skewness or kurtosis exceeds 1 we will consider them non-normal and p-values will be validated using a bootstrap method (see Bootstrapping, below).

1. **Bootstrapping**

Bootstrapping of standard errors will be applied for any non-normal outcome variable and p-values and 95% confidence intervals derived using these. To enable bootstrapping we will use Stata to generate a set of pseudorandom numbers to act as seeds for each bootstrap analysis; providing there are enough non-normal outcomes to justify this.

1. **Choice of covariates**

The covariates to be included in all primary and secondary analyses will be selected as described below.

A pre-specified group of covariates will be included as listed in Table 1. This list includes the study “design factors”, used as part of the stratification procedure for randomising allocation to trial arms (hospital site and gender), plus two variables considered prognostic of the outcomes: age and current anxiety/depression medication.

Table 2 lists additional available covariates. We will look for imbalance between trial arms on each of these and include those with a standardised mean difference between arms of >0.25 in the primary analysis model. Some categories within variables may be collapsed; the choices here will be determined by descriptive analysis of baseline values prior to any endpoint analysis.

| **Table 1** | |
| --- | --- |
| **Pre-specified covariates** | **Description** |
| Gender (design factor) | Two levels |
| Hospital site (design factor) | Categorical |
| Age | Continuous |
| Medication for depression or anxiety | Never taken/Currently taking/Taken in the past |

| **Table 2** | | |
| --- | --- | --- |
| **Additional available covariates** | **Description** | |
| Highest qualification | Categorical |  |
| BMI | Continuous | |
| Ethnicity | Categorical | |
| Marital status | Two levels: Married/in relationship versus single | |
| Time from baseline to measurement (in days) | Continuous | |
| Employment status | Categorical | |
| Smoking status | Never/Passive/Ex/Smoker | |
| Alcohol units per month | Continuous | |
| Age at first cardiovascular event | 0-18, 19-24, 25-33, 34-44, 45-54, 55-64, 65 or older | |
| Number of co-morbidities | Continuous | |
| Number of different previous cardiovascular event | Continuous | |
| Psychological therapies for anxiety or depression | Never received/Currently receiving/Received in the past | |

**NAME: David Reeves (study lead statistician)**


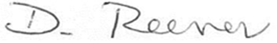


Signature…………………………………………….. Date…24/03/2021……………

**Name: Adrian Wells (Chief Investigator)**

A. Wells

Signature…………………………………………….. Date……24/03/2021…………

**Name: Kate Jolly (Chair of Steering Committee)**

Signature agreed via email Date… April 29, 2021.
